# An Osteoblast-Derived Proteinase Controls Tumor Cell Survival via TGF-beta Activation in the Bone Microenvironment

**DOI:** 10.1371/journal.pone.0029862

**Published:** 2012-01-04

**Authors:** Sophie Thiolloy, James R. Edwards, Barbara Fingleton, Daniel B. Rifkin, Lynn M. Matrisian, Conor C. Lynch

**Affiliations:** 1 Department of Cancer Biology, Vanderbilt University, Nashville, Tennessee, United States of America; 2 Nuffield Orthopaedic Centre, University of Oxford, Oxford, United Kingdom; 3 Department of Cell Biology, New York University School of Medicine, New York, New York, United States of America; 4 Tumor Biology Department, H. Lee Moffitt Cancer Center, Tampa, Florida, United States of America; Stanford University, United States of America

## Abstract

**Background:**

Breast to bone metastases frequently induce a “vicious cycle” in which osteoclast mediated bone resorption and proteolysis results in the release of bone matrix sequestered factors that drive tumor growth. While osteoclasts express numerous proteinases, analysis of human breast to bone metastases unexpectedly revealed that bone forming osteoblasts were consistently positive for the proteinase, MMP-2. Given the role of MMP-2 in extracellular matrix degradation and growth factor/cytokine processing, we tested whether osteoblast derived MMP-2 contributed to the vicious cycle of tumor progression in the bone microenvironment.

**Methodology/Principal Findings:**

To test our hypothesis, we utilized murine models of the osteolytic tumor-bone microenvironment in immunocompetent wild type and MMP-2 null mice. In longitudinal studies, we found that host MMP-2 significantly contributed to tumor progression in bone by protecting against apoptosis and promoting cancer cell survival (caspase-3; immunohistochemistry). Our data also indicate that host MMP-2 contributes to tumor induced osteolysis (μCT, histomorphometry). Further *ex vivo*/*in vitro* experiments with wild type and MMP-2 null osteoclast and osteoblast cultures identified that 1) the absence of MMP-2 did not have a deleterious effect on osteoclast function (cd11B isolation, osteoclast differentiation, transwell migration and dentin resorption assay); and 2) that osteoblast derived MMP-2 promoted tumor survival by regulating the bioavailability of TGFβ, a factor critical for cell-cell communication in the bone (ELISA, immunoblot assay, clonal and soft agar assays).

**Conclusion/Significance:**

Collectively, these studies identify a novel “mini-vicious cycle” between the osteoblast and metastatic cancer cells that is key for initial tumor survival in the bone microenvironment. In conclusion, the findings of our study suggest that the targeted inhibition of MMP-2 and/or TGFβ would be beneficial for the treatment of bone metastases.

## Introduction

Breast to bone metastasis is a common event during breast cancer progression with the resultant lesions typically hallmarked by extensive areas of bone destruction [1]. Despite medical advances, breast to bone metastases are incurable with treatments being mainly palliative [2], [3]. Only by elucidating the molecular mechanisms through which breast cancer cells interact with host cells of the bone microenvironment can new therapies be generated.

Metastatic breast cancer cells induce osteolytic lesions by high-jacking the normal bone remodeling process; a finely regulated biological event comprised of osteoblast mediated bone synthesis coupled with osteoclast mediated bone resorption [4]. Our current understanding of the mechanisms underlying tumor-induced osteolysis is best encapsulated by the concept of the ‘vicious cycle’; a cycle in which metastatic breast cancer cells secrete factors, such as parathyroid hormone related peptide (PTHrP) [5], that in turn stimulate osteoblast expression of factors including receptor activator of nuclear kappa B ligand (RANKL) that promote osteoclast recruitment and activation [6]. Osteoclasts mediate bone destruction by; forming a resorptive seal on the surface of mineralized bone, lowering the pH to promote de-mineralization and, secreting cathepsin-K, an acidophilic type I collagenase into the resorption lacunae [7]. Osteoclast mediated bone resorption results in the liberation and activation of growth factors such as transforming growth factor β (TGFβ) that are sequestered in the bone matrix [8]. The release of these stored factors in turn can promote the growth of the tumor cells, thereby completing the vicious cycle [3]. Osteoblasts are a critical intermediate between the metastatic breast cancer cells and the osteoclasts and are therefore essential for the forward momentum of the vicious cycle. However, little information is available as to whether osteoblasts can impact tumor behavior directly *in vivo*.

Prior to osteoclast resorption of the mineralized bone matrix, bone lining osteoblasts must remodel the non-mineralized osteoid canopy and retract from the bone surface [9]. The retraction step requires osteoblast derived proteolytic activity [10], [11]. Surprisingly, despite their involvement in the generation of bone matrix, osteoblasts express a number of proteinases including matrix metalloproteinases (MMPs) [12], [13], [14]. While MMPs can degrade numerous components of the extracellular matrix (ECM), recent studies have implicated MMPs as important mediators of cell-cell communication by virtue of their ability to process multiple non-matrix molecules, such as cytokines and growth factors, to soluble forms that have either enhanced or attenuated activities [15]. In assessing MMP expression in human breast-to-bone metastases and in a mouse model of the osteolytic tumor-bone microenvironment, we found that MMP-2 was largely localized to osteoblasts.

Given that osteoblasts express MMP-2 and that MMP-2 is important for osteoblast function [14], [16], we tested whether this osteoblast derived proteinase impacted the osteolytic vicious cycle. Surprisingly, we observed that host MMP-2 did not impair osteoclast behavior but that osteoblast derived MMP-2 was critical for tumor survival in the bone microenvironment via a mechanism involving the activation of latent TGFβ. Our findings suggest the presence of a mini-vicious cycle between osteoblasts and the metastatic cancer cells in the osteolytic tumor-bone microenvironment that is not dependent on osteoclast activity.

## Results

### Osteoblasts express MMP-2 in the human and murine osteolytic tumor-bone microenvironment

**Figure 1:**
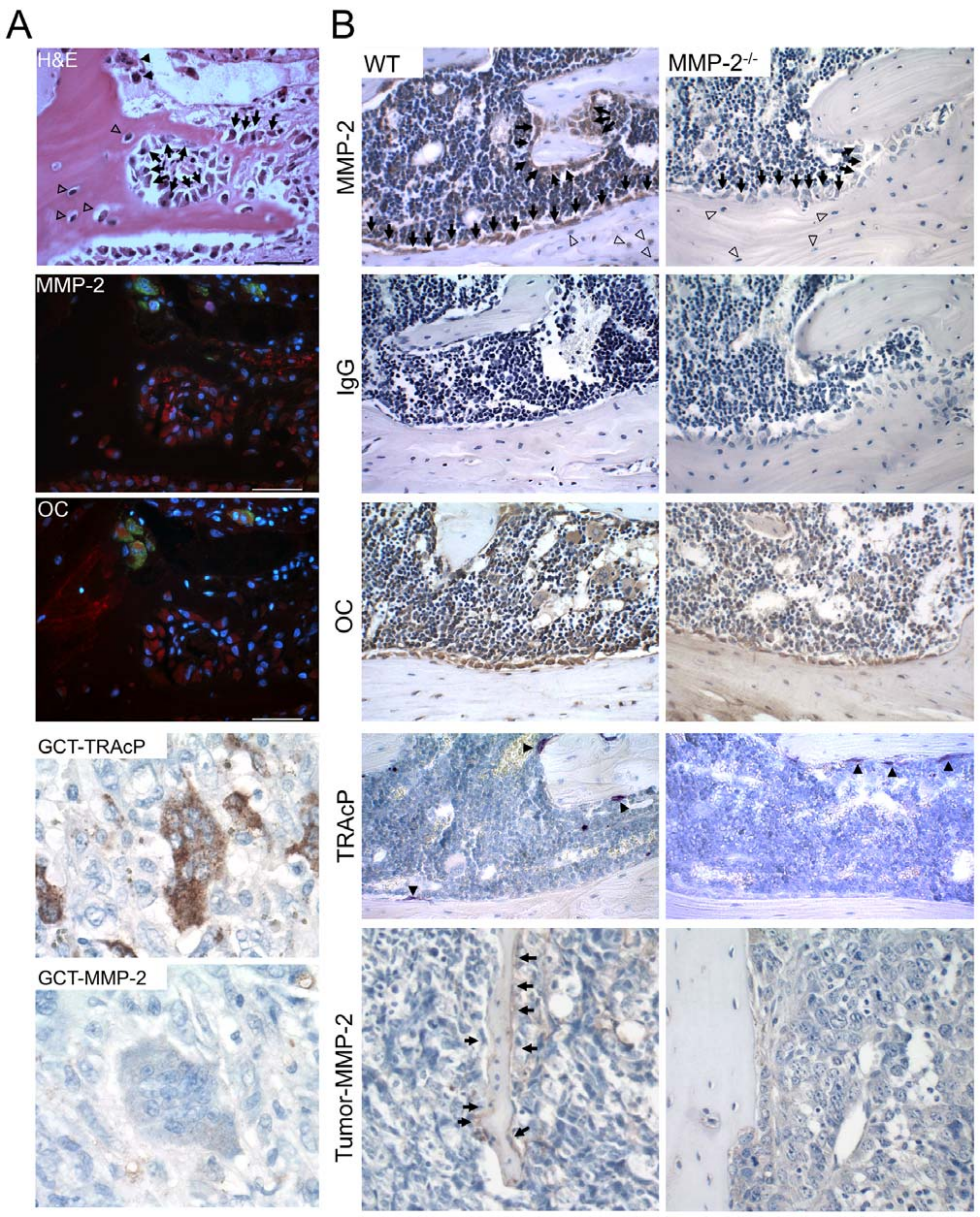
Localization of MMP-2 in the human and murine bone microenvironment. **A**, Fluorescent TRAcP staining (green) was used to localize osteoclasts (closed arrow heads) while immunofluorescence was used to localize osteoblast marker osteocalcin and MMP-2 (red) in human samples of breast to bone metastasis (n = 11). DAPI (blue) was used as a nuclear stain. H&E was used to identify tumor-bone morphology. Arrows indicate osteoblasts while open arrow heads indicate osteocytes. Scale bars represent 50 µm. The localization of MMP-2 in human GCT osteoclasts (TRAcP positive, brown) was also assessed. **B**, Immunohistochemistry for MMP-2 (brown) and osteocalcin (OC) in the wild type and MMP-2 null murine bone and tumor-bone microenvironment. Rabbit IgG was used as a negative control. Arrows indicate osteoblasts while open arrow-heads indicate osteocytes. Colorimetric TRAcP staining (red) was also used to identify osteoclasts (arrow heads).

Using a rodent model, we previously identified that MMPs were highly expressed in the tumor-bone microenvironment with subsequent studies revealing that MMPs such as MMP-7 and MMP-9 were largely localized to osteoclasts in this setting [17], [18]. In contrast, analysis of MMP-2 expression revealed that MMP-2 was localized to both the tumor and stroma of human (n = 11) and murine osteolytic bone metastases (Fig. 1A–B). Of note, osteoblasts and osteocytes were found to be consistently positive for MMP-2 in human samples and in the control and tumor bearing limbs of the wild type mice but surprisingly, human and murine osteoclasts were largely negative for MMP-2 (Fig. 1A–B). Although other stromal components were positive for MMP-2 we focused our attention on the osteoblast compartment given their critical role as an intermediate in the vicious cycle and reports documenting the contribution of osteoblast derived MMP-2 to bone development [14], [16]. Therefore, we next tested the impact of host MMP-2 ablation on this process in an immunocompetent model of mammary tumor induced osteolysis.

### Host MMP-2 significantly impacts tumor survival in the bone microenvironment

**Figure 2:**
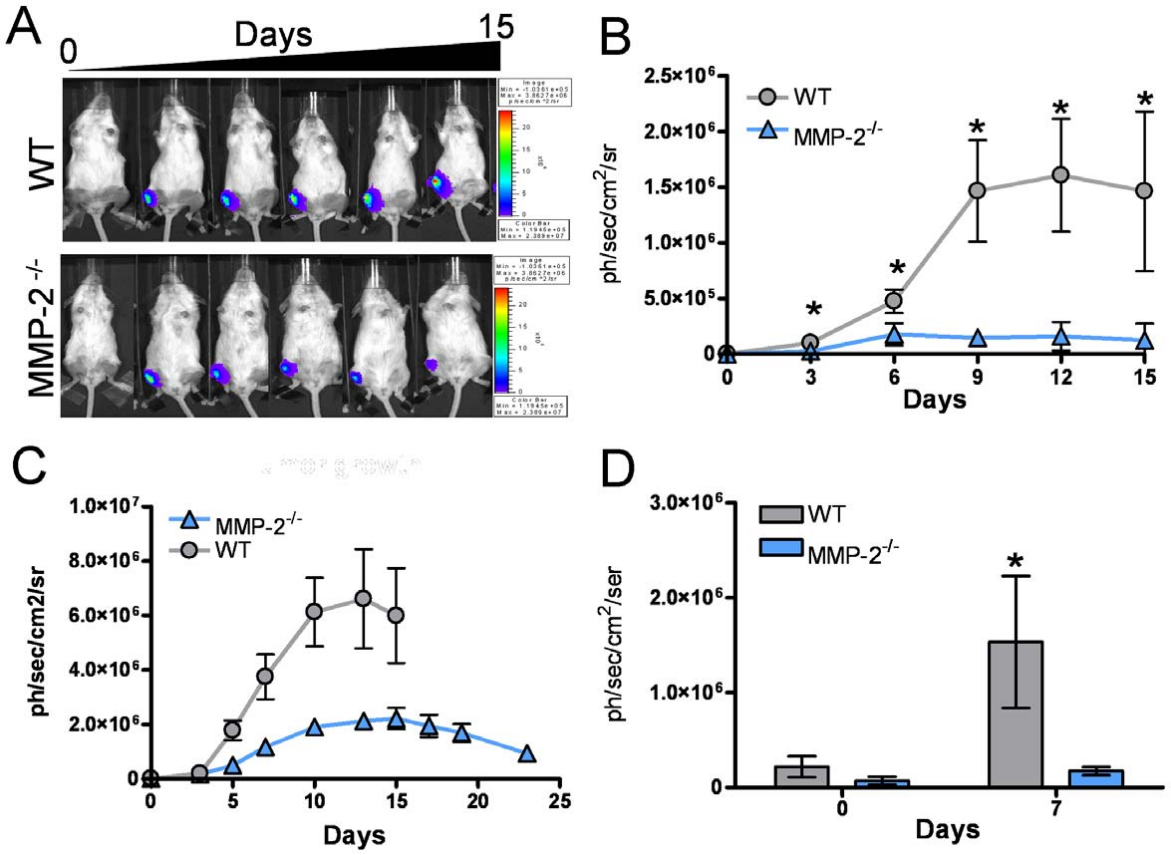
Host-derived MMP-2 impacts mammary tumor growth in the bone microenvironment. **A**, Representative timeline of PyMT-Luc luciferase expression in FVB wild type (WT; n = 10) and MMP-2 null (MMP-2^−/−^; n = 10) mice. Hotter colors indicate areas of increased luciferase activity. **B**, Graph represents PyMT-Luc tumor growth over time as assessed by luciferase activity in WT and MMP-2^−/−^ animals. **C**, PyMT-Luc fail to grow in the MMP-2^−/−^ bone microenvironment as assessed over a 25 day period. PyMT-Luc were injected intratibially into syngeneic FVB wild type (WT; n = 10) or MMP-2 null (MMP-2^−/−^; n = 10) mice. WT mice were euthanized on day 15 due to tumor size. **D**, 17L3C-Luc, an unrelated cell line derived from the PyMT model of mammary gland tumorigenesis, was intratibially injected into wild type (WT; n = 5) or MMP-2 null (MMP-2^−/−^; n = 5) mice. Luciferase activity was assessed as a measure of tumor growth over a 7-day period. Data are mean ± SD; Asterisk denotes statistical significance (p<0.05).

To determine the contribution of host derived MMP-2 in mammary tumor growth in bone, two independent mammary tumor cell lines derived from the transgenic polyoma middle T antigen (PyMT) model of mammary tumorigenesis, denoted PyMT-Luc and 17L3C-Luc [19], [20], were injected into the tibia of six week old syngeneic immunocompetent FVB wild type (n = 10) and MMP-2 null (n = 10) animals. Upon intratibial injection, luciferase activity was recorded over time. Quantitation of the bioluminescent signal from the PyMT-Luc tumor cells showed a marked decrease in tumor growth rate in MMP-2 null mice compared to wild type controls from day 3 post-injection onwards (Fig. 2A–B). Tumors in the MMP-2 null mice were imaged for at least 25 days and we observed that the bioluminescent signal never reached the level obtained in the wild type mice at day 9 (Fig. 2C). These data suggested that host MMP-2 was important for the initial survival and establishment of tumor cells in the bone. The observed effect of MMP-2 on tumor growth was confirmed using the unrelated PyMT derived cell line, 17L3C-Luc (Fig. 2D). These experiments were repeated on five independent occasions with similar sized groups (n≥10 per group) and similar observations were recorded.

**Figure 3:**
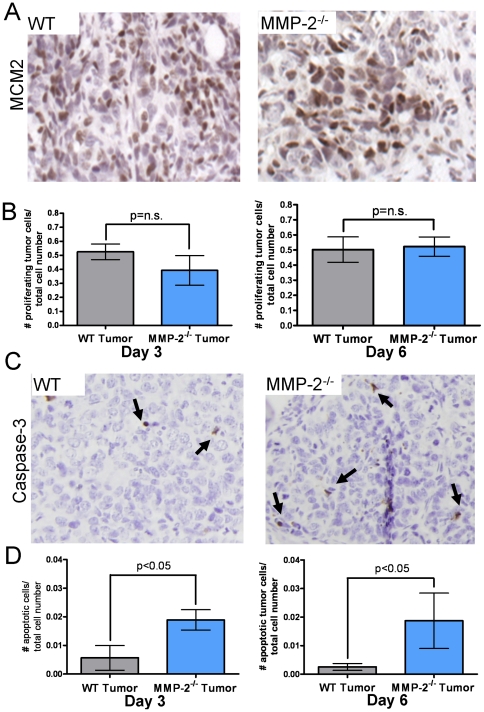
Host-derived MMP-2 impacts tumor survival in the bone microenvironment. **A**, Representative photomicrograph of MCM2 positive proliferating (brown) cells in the wild type (WT) and MMP-2 null (MMP-2^−/−^) tumor-bone microenvironment. **B**, Proliferation in the tumor-bone microenvironment as a function of total cell number was assessed by staining for Mcm2 in tumor bearing tibias of WT and MMP-2 null mice at day 3 and day 6 post-surgery. **C**, Representative photomicrograph of caspase-3 positive apoptotic (brown) cells in the wild type (WT) and MMP-2 null (MMP-2^−/−^) tumor-bone microenvironment. **D**, Apoptosis in the tumor-bone microenvironment as a function of total cell number was assessed by staining for cleaved caspase-3 at day 3 and day 6 post surgery. Data are mean ± SD; n.s. implies a non-significant p value (p>0.05).

The impact of host MMP-2 on mammary tumor growth in the bone was analyzed by immunohistochemical staining for Mcm2 (proliferation) and cleaved caspase-3 (apoptosis) at the day 3 time point since this was consistently the first time point when tumor growth differences were noted between the wild type and MMP-2 null animals. Surprisingly, no difference in tumor proliferation was observed between the two groups either at day 3 or at day 6 (Fig. 3A–B). However, in comparison to wild type controls, MMP-2 null mice showed a significantly higher level of apoptotic tumor cells at day 3 and this difference persisted to day 6 (Fig. 3C–D). These data demonstrate for the first time that host MMP-2 impacts tumor growth in the bone microenvironment by promoting tumor cell survival.

### Host MMP-2 contributes to tumor induced osteolysis

**Figure 4:**
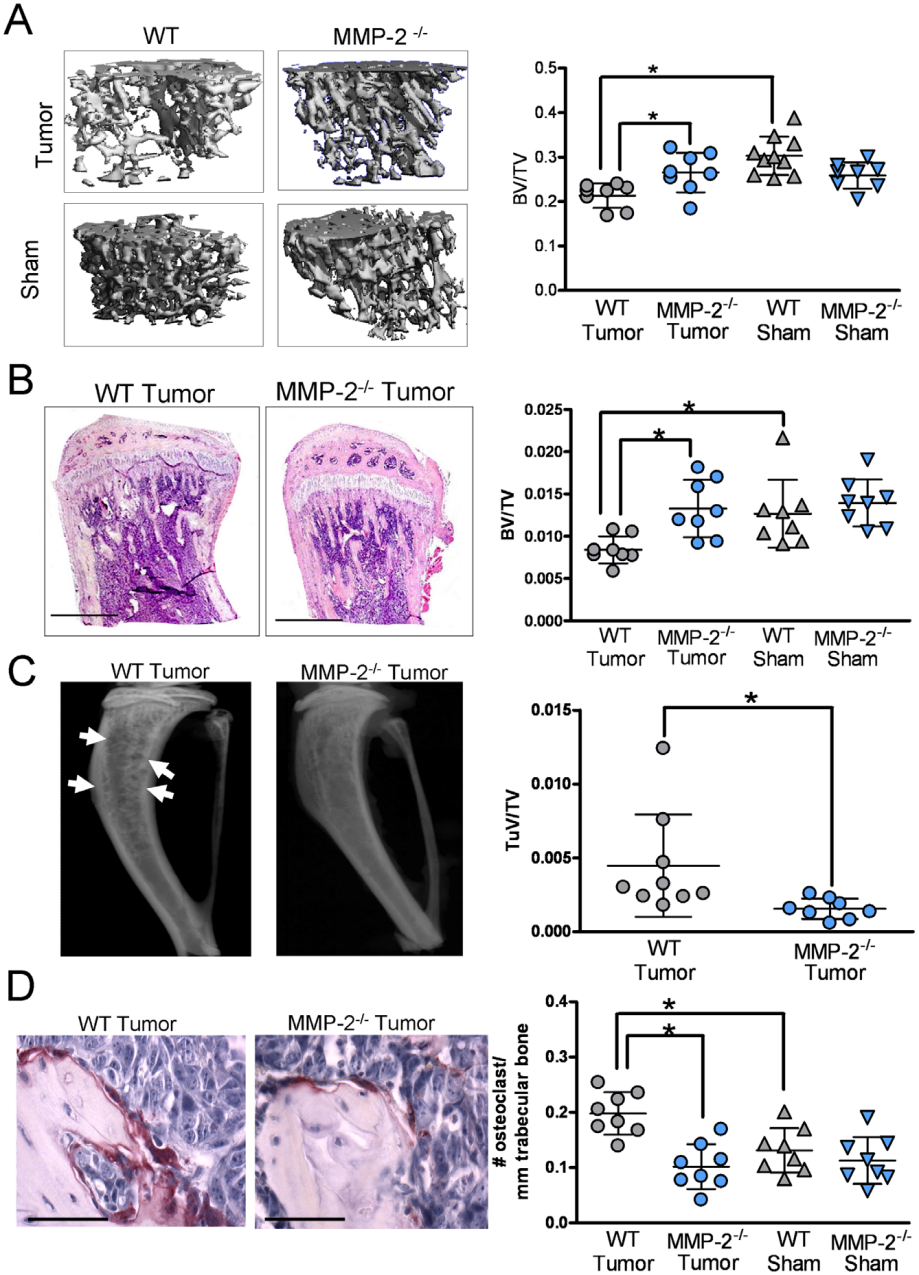
Tumor induced osteolysis is controlled by host MMP-2. **A**, Representative μCT scans of trabecular bone from tumor bearing and sham injected limbs of wild type (WT) and MMP-2 null (MMP-2^−/−^) mice at day 9. Graph illustrates ratio of bone volume to total volume (BV/TV). **B**, Representative H&E stained photomicrographs of tumor bearing tibias from WT and MMP-2^−/−^ mice. Scale bars represent 1 mm. Graph represents the BV/TV ratio. **C**, Representative radiographic images from tumor injected WT and MMP-2^−/−^ animals at day 9. Arrow indicates lytic tumor lesions in the wild type group. Graph represents the tumor volume (TuV) over total volume (TV) ratio. **D**, Representative TRAcP stained photomicrographs of tumor injected WT and MMP-2^−/−^ animals at day 9. Scale bar represents 50 µm. Graph indicates the number of osteoclasts/mm of trabecular bone. Data are mean ± SD. Asterisk denotes that p<0.05.

The vicious cycle paradigm dictates that increased tumor growth leads to increased bone resorption and vice versa. Since decreased tumor growth was observed in MMP-2 null mice, we next assessed whether there was a concomitant decrease in osteolysis in the MMP-2 null tumor-bone microenvironment. Of note, MMP-2 null mice display transient bone phenotypes during skeletal development [14]. However, analysis of baseline trabecular bone volume by high resolution μCT revealed no differences between the wild type and MMP-2 null mice at 6 weeks of age (data not shown). μCT and histomorphometry analyses of the trabecular bone content was performed on wild type and MMP-2 null mice at the end of the study period (day 9). Tumor bearing limbs of wild type mice showed a significant decrease in the trabecular bone volume compared to the MMP-2 null group by μCT (Fig. 4A) and by histomorphometry (Fig. 4B). No differences were detected between wild type and MMP-2 null sham injected control limbs (Fig. 4A–B). Decreased bone resorption in the MMP-2 null tumor bearing group compared to wild type controls was further supported by X-ray radiography analysis and by the number of mature multinucleated TRAcP positive bone lining osteoclasts (Fig. 4C–D). These results implicate a role for host derived MMP-2 in mediating mammary tumor induced osteolysis.

### MMP-2 deficiency does not inhibit osteoclast precursor migration or osteoclastogenesis

**Figure 5:**
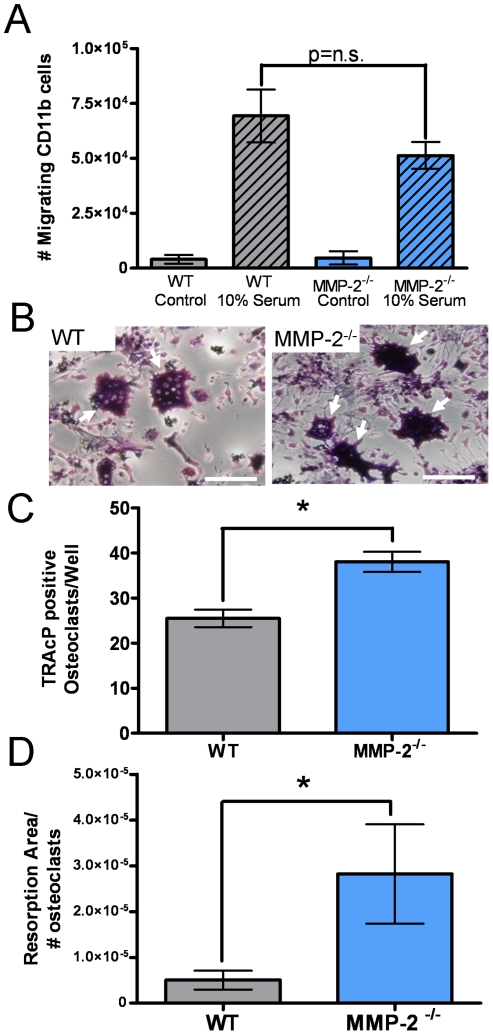
Absence of host MMP-2 does not impair osteoclast precursor function. **A**, Quantitative analysis of isolated wild type (WT) and MMP-2 null (MMP-2^−/−^) CD11b^+ve^ osteoclast precursor migration to 10% serum or control serum free media. **B**, Analysis of the osteoclastogenic capability of WT or MMP-2^−/−^ osteoclast precursors. Arrows indicate TRAcP positive (red) multinucleated (blue) osteoclasts. Scale bars represent 50 µm. **C**, Analysis of the number of WT and MMP-2^−/−^ mature osteoclasts formed/well in each group (n≥3/group). **D**, Pit formation assay on dentin discs to test the functionality of mature WT and MMP-2^−/−^ osteoclasts. Asterisk denotes that p<0.05 while p = n.s. denotes non-significant p values.

Although mature osteoclasts were largely negative for MMP-2 expression by immunohistochemistry (Fig. 1), it is possible that MMP-2 may be expressed in early osteoclast precursors and therefore, MMP-2 could impact mammary tumor growth-induced osteolysis by affecting a) migration/recruitment of osteoclast precursors and/or b) osteoclastogenesis. To address this, CD11b^+ve^ myeloid/osteoclast precursors were isolated from the long bones of wild type and MMP-2 null animals. Migration assays using 10% serum revealed no difference between the wild type and MMP-2 null osteoclast precursors (Fig. 5A). Surprisingly, osteoclastogenesis and resorption assays using an equal number of starter precursor cells revealed MMP-2 null osteoclasts consistently generated a significantly higher number of functional multinucleated osteoclasts compared to the wild type controls (Fig. 5B–D). The higher numbers of osteoclasts generated by the MMP-2 null osteoclast precursors was unexpected given that less mature bone resorbing osteoclasts were identified in the tumor-bone microenvironment of the MMP-2 null mice (Fig. 4D). Importantly however, our *in vitro* data, demonstrated that the function of MMP-2 null osteoclasts was not compromised. Therefore, we hypothesized that the decreased tumor survival in the MMP-2 null tumor-bone microenvironment may be osteoblast mediated, especially given their consistent positivity for MMP-2 expression in the tumor-bone microenvironment (Fig. 1).

### Osteoblast-derived MMP-2 mediates tumor survival

**Figure 6:**
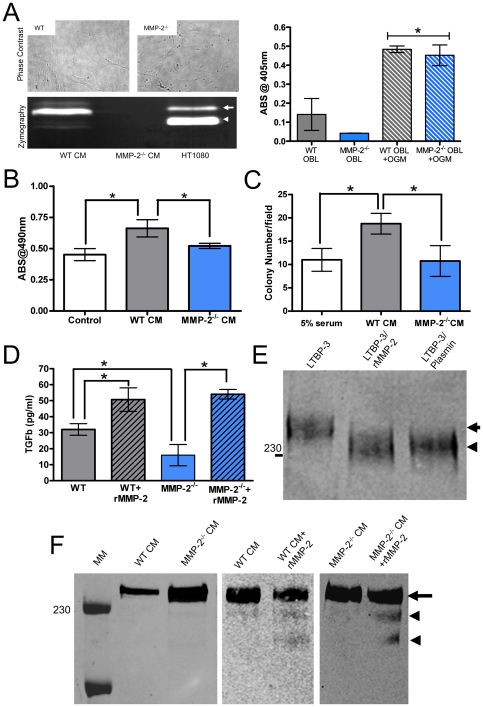
Osteoblast-derived MMP-2 controls TGF-β bioavailability. **A**, Representative phase contrast photomicrographs of isolated wild type (WT) and MMP-2 null (MMP-2^−/−^) primary osteoblast morphology. Osteoblasts were characterized by differentiation in the presence or absence of OGM and measuring alkaline phosphatase activity. Asterisk denotes statistical significance (p<0.05) between the OGM and the respective control treated groups. Conditioned media derived from wild type (WT CM) and MMP-2 null (MMP-2^−/−^ CM) primary osteoblasts was also assessed for the presence of MMP-2 using gelatin zymography. Arrow denotes latent MMP-2 while arrow head denotes active MMP-2. HT1080, a human firbosarcoma cell line was used as a positive control for MMP-2. **B** and **C**, PyMT-Luc cells were treated with conditioned media from wild type (WT CM) or MMP-2 null (MMP-2^−/−^ CM) osteoblasts. The metabolic activity of PyMT-Luc cells treated with WT CM or MMP-2^−/−^ CM was assessed by MTT assay (**B**) and survival was determined by soft agar colony formation (**C**). **D**, Levels of TGFβ in WT and MMP-2^−/−^CM were assessed by ELISA. Changes in TGFβ levels were also assessed after the addition of recombinant MMP-2 (rMMP-2; 100 ng/ml CM for 3 hours at 37°C). **E**, The ability of MMP-2 (100 ng recombinant MMP-2/ml CM 3 hours at 37°C) to process LTBP-3 derived from transfected COS-7 cells was assessed by immunoblot as described [32]. Plasmin (1 µg/ml of CM 1 hour at 37°C) was used as a positive control. **F**, LTBP-3 levels and the impact of recombinant MMP-2 on LTBP-3 processing in the CM derived from WT and MMP-2^−/−^ osteoblasts was determined by immunoblot analysis. MW is indicated by 230 kDa. Arrow indicates full length LTBP-3.

Since osteoblasts express MMP-2 in the tumor-bone microenvironment and given our data suggesting host derived MMP-2 was impacting tumor survival *in vivo* (Fig. 3C–D), we next tested the impact of wild type and MMP-2 null primary osteoblasts on tumor survival *in vitro*. Characterization studies of the isolated wild type and MMP-2 null osteoblasts revealed no significant morphological or functional differences with respect to differentiation (Fig. 6A). Zymography analysis of conditioned media derived from the wild type and MMP-2 null osteoblast cultures also demonstrated the presence of latent and active MMP-2 in the wild type cultures only (Fig. 6A).

**Figure 7:**
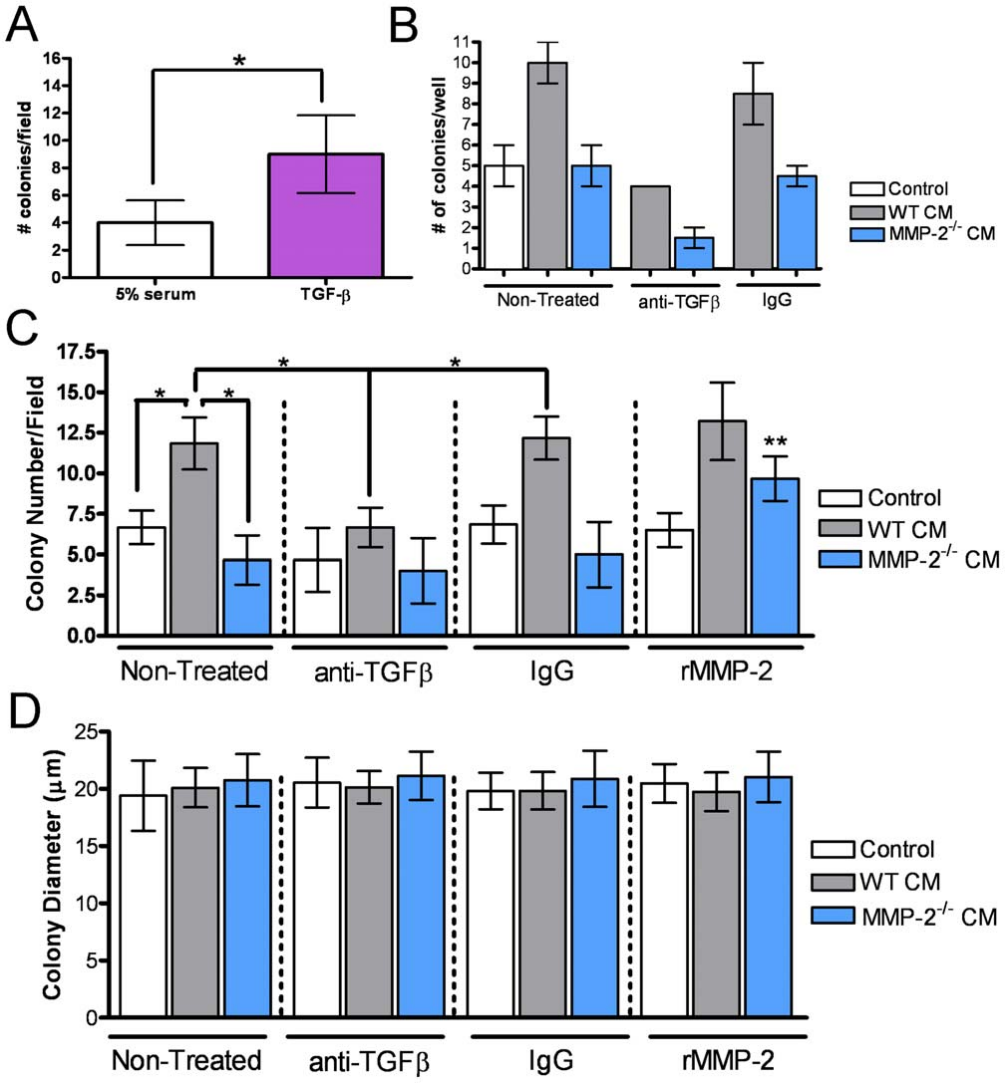
MMP-2 mediates tumor survival in a TGFβ dependent manner. **A**, Tumor survival in the presence of 1 ng/ml recombinant TGFβ was assessed by soft agar colony formation assay. **B**, The number of PyMT-Luc colonies formed in response to 10 days of treatment with control media (5% serum containing αMEM) or conditioned media derived from wild type or MMP-2 null osteoblasts was analyzed in a 2D (plastic) assay (non-treated group). Under similar conditions the impact of TGFβ inhibition was also determined (matched isotype antibody was added to control wells). Differences are significant as assessed by ANOVA. **C**, The effects of blocking TGFβ in the CM derived from WT and MMP-2^−/−^ osteoblasts was determined by soft agar colony formation assay. Data are mean ± SD. Asterisk indicates p<0.05 and n.s indicates non significance. Double asterisk denotes that the addition of recombinant MMP-2 to MMP-2^−/−^ CM significantly enhances tumor survival compared to MMP-2^−/−^ CM alone. **D**, Osteoblast-derived MMP-2 and TGF-β mediate tumor survival without impacting colony size. Using a soft agar colony formation assay, no difference in colony size was detected between the various treatment conditions. Data are mean ± SD.

Next, we assessed the ability of conditioned media from wild type and MMP-2 null primary osteoblast cultures to modulate PyMT-Luc cell growth and survival *in vitro* using MTT and soft agar colony formation assays. We found that conditioned media derived from wild type primary osteoblasts resulted in significantly higher metabolic activity and in a higher number of tumor colonies compared to tumor cells incubated with conditioned media from MMP-2 null osteoblasts (Fig. 7B–C). These data suggest that an osteoblast derived proteinase, MMP-2, could influence tumor survival; a conclusion that was in agreement with our *in vivo* studies (Fig. 3).

Subsequently, we explored the potential molecular mechanisms through which osteoblast derived MMP-2 could control tumor survival. Analysis of the literature revealed TGFβ to be a strong candidate since; a) TGFβ is incorporated into the bone matrix and osteoid canopy by osteoblasts and has been identified as a master regulator of the vicious cycle via it's effects on the behavior of the tumor cells, the osteoblasts themselves and the osteoclasts [21]; b) MMP-2 has been shown to mediate the processing of the proteins that sequester TGFβ in a latent state, namely the latency associated peptide (LAP) and the latency binding proteins (LTBP) -1 and -4 [22], [23], [24], [25], [26] and; c) while TGFβ has pleiotropic effects, it has been shown to impact tumor survival in the mammary gland by protecting against apoptosis and bone marrow progenitor survival in the bone microenvironment by abrogating the effects of Fas ligand (FasL) [27], [28].

Initially we examined the levels of TGFβ by ELISA in the conditioned media derived from the osteoblast primary cultures. We found significantly higher levels of TGFβ in the conditioned media derived from the wild type osteoblasts compared to the levels detected in the conditioned media of the MMP-2 null osteoblasts and further, that the level of active TGFβ could be significantly enhanced via the addition of recombinant MMP-2 (Fig. 6D). *In vivo*, TGFβ is maintained in a latent form via its complex with the latency associated peptide (LAP) and members of the latent TGFβ binding protein family (LTBP-1-4). Successive proteolytic cleavages are required in order to generate active TGFβ [29], [30]. MMP-2 has previously been identified as cleaving LAP and LTBPs-1 and 4. Interestingly, of the LTBP proteins, only LTBP-3 has been implicated in bone development since LTBP-3 null mice display a distinct cranial phenotype and develop osteopetrosis [31], [32], [33], [34]. Therefore, we tested whether MMP-2 was capable of processing LTBP-3. Conditioned medium of COS-7 cells overexpressing the large latent complex of LTBP-3 and LAP-TGFβ was subjected to digestion with recombinant active MMP-2. The molecular weight of the complex was reduced from ∼240 kDa to ∼230-220 kDa in the presence of recombinant active MMP-2, a processing event that is consistent with that observed for plasmin (Fig. 6E) [35]. Next, we analyzed LTBP-3 in conditioned media derived from wild type and MMP-2 null primary osteoblast cultures. A comparison of conditioned media, normalized for total protein content, identified higher levels of LTBP-3 in the conditioned media derived from the MMP-2^−/−^ osteoblasts compared to the wild type control (Fig. 6F). Furthermore, the addition of recombinant MMP-2 (100 ng/ml for one hour at 37°C) to conditioned media (equally loaded for LTBP-3 using densitometry) demonstrated that LTBP-3 could be processed further (Fig. 6F). These data suggest LTPB-3 is an MMP-2 substrate, and support our hypothesis that osteoblast derived MMP-2 can mediate the activation of TGFβ.

### Osteoblast derived MMP-2 mediates tumor survival via TGFβ

To test whether TGFβ could mediate tumor survival, we initially used a soft agar colony formation assay and found that treatment of the tumor cells with recombinant active TGFβ significantly increased the number of colonies compared to control conditions (Fig. 7A). To test whether TGFβ was the principal molecule in the osteoblast conditioned media through which MMP-2 impacted tumor survival, we utilized a TGFβ neutralizing antibody (2G7). Addition of the neutralizing TGFβ antibody to the conditioned media harvested from wild type osteoblasts significantly reduced tumor survival compared to IgG controls in 2D (plastic) and soft agar colony formation assays (Fig. 7B–C). We observed that the addition of the neutralizing TGFβ antibody to the MMP-2 null osteoblast conditioned media had no impact on tumor survival (Fig. 7B–C), while the addition of recombinant MMP-2 to conditioned media from MMP-2 null primary osteoblasts rescued the tumor survival phenotype (Fig. 7C). In the colony formation assays, no difference was observed in the average size of the colonies, suggesting that the absence of MMP-2 in osteoblasts affects tumor survival but not tumor proliferation, a conclusion that is in agreement with our *in vivo* data (Fig. 7D). For the first time, these data demonstrate that an osteoblast derived proteinase, MMP-2, can impact tumor survival.

### Host MMP-2 impacts TGFβ bioavailability and tumor survival *in vivo*

**Figure 8:**
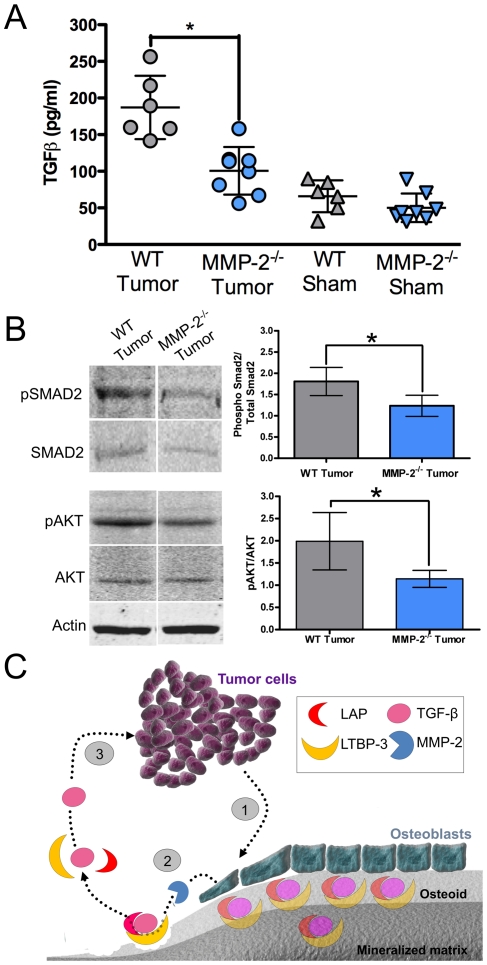
Osteoblast-derived MMP-2 impacts TGFβ activation and tumor survival in the *in vivo* tumor-bone microenvironment. **A**, The levels of TGFβ in normalized tumor-bone lysates derived from WT and MMP-2^−/−^ tumor or sham injected tibias was assessed by ELISA. **B**, Representative immunoblots for phospho-SMAD (pSMAD2), total smad2 (SMAD2), phospho-AKT (pAKT), total AKT (AKT) and actin (loading control) in the tumor-bone lysates derived from WT and MMP-2^−/−^ mice. Densitometry on immunoblots generated from tumor bone lysates of at least 5 animals per group was used to generate graphs of the ratio of pSMAD2/SMAD2 and pAKT/AKT. Data are mean ± SD. *, p<0.05. **C**, Osteoblast derived MMP-2 controls TGFβ activation and mammary survival in the tumor-bone microenvironment. (**1**) Tumor derived signals induce osteoblast retraction from mineralized matrix and the resorption of the non-mineralized osteoid canopy. (**2**) We posit that MMP-2 processing of the factors that sequester TGFβ in a latent state such as LTBP-3 and LAP initiates TGFβ activation. (**3**) Our data show that the activation of TGFβ by osteoblast derived MMP-2 mediates tumor survival and we hypothesize this mini-vicious cycle is essential for the establishment of the traditional vicious cycle of tumor induced osteolysis.

Having demonstrated that osteoblast derived MMP-2 mediated the activation of TGFβ and tumor survival *in vitro*; we determined the relevance of the mechanism in the *in vivo* osteolytic tumor-bone microenvironment. We found that lysates, normalized for total protein content, derived from the wild type tumor injected tibias had significantly higher levels of active TGFβ compared to the MMP-2 null tumor bone lysates (Fig. 8A). Further, analysis of downstream TGFβ signaling revealed that the ratio of phospho-smad2 to total smad2 was also significantly higher in the wild type tumor-bone lysates compared to the MMP-2 null tumor bone lysates (Fig. 8B). We also examined the levels of phospho-AKT as a general readout for cell survival in the tumor bone microenvironment [27]. Consistent with our conclusion that TGFβ was promoting cell survival, we observed significantly higher ratios of phospho AKT to total AKT in the tumor-bone lysates derived from the wild type mice compared to the MMP-2 null mice (Fig. 8B). Collectively, these data support our overarching hypothesis that an osteoblast derived proteinase, MMP-2, is key for mediating for TGFβ activation and tumor survival *in vivo*. Furthermore, our studies reveal a novel interplay between the tumor cells and osteoblasts that is independent of the osteoclast compartment and suggests the presence of a mini-vicious cycle in the tumor-bone microenvironment that is critical for initial tumor survival and establishment (Fig. 8C).

## Discussion

Breast to bone metastasis is an incurable disease that commonly affects women with late stage breast cancer [1]. Lytic bone lesions cause severe complications that greatly impact the patient's quality of life. Surgery, radiotherapy and chemotherapy with bisphosphonates are tools currently employed to tackle the disease yet these treatments are mainly palliative rather than curative [2], [3]. Therefore, identifying the molecular mechanisms underlying cell-cell communication in the tumor-bone microenvironment is key for the development of therapies that can treat and ultimately cure the disease.

### The osteoblast-tumor mini-vicious cycle is mediated by MMP-2 and TGFβ

To date, the majority of studies examining the breast to bone metastatic microenvironment have focused on the final step of the vicious cycle, i.e. how osteoclasts are recruited and activated to the tumor-bone microenvironment to induce bone destruction. Tumor stimulation of osteoblasts to secrete osteoclastogenic factors is critical in mediating osteoclastogenesis in order to complete the vicious cycle [3]. However, little emphasis has been placed on whether the osteoblasts themselves can impact tumor behavior in the *in vivo* bone-microenvironment. Our studies show for the first time that an osteoblast derived proteinase, MMP-2, can significantly impact on tumor survival and establishment in the mammary tumor-bone microenvironment. Furthermore, we suggest that MMP-2 processing of the factors that sequester TGFβ in a latent state is the principal mechanism underlying our observations. Based on these data, we posit the existence of a vicious mini-cycle in the context of the larger osteolytic vicious cycle in which the osteoblast is critical for mediating the survival and establishment of the tumor cells in the bone microenvironment (Fig. 8C). Our observations support this conclusion since; a) tumor growth is significantly attenuated at an early time point in MMP-2 null animals (Fig. 2–3); b) the absence of MMP-2 does not negatively impact osteoclast migration or function (Fig. 5); c) conditioned media derived from the MMP-2 null osteoblasts failed to promote tumor survival compared to conditioned media from wild type osteoblasts (Fig. 6–7); d) the addition of exogenous MMP-2 to the MMP-2 null osteoblasts resulted in an increase in active TGFβ that subsequently promoted tumor survival (Fig. 6) and; e) the use of a TGFβ neutralizing antibody blocked the survival effect observed with the wild type osteoblast conditioned media (Fig. 7).

MMP-2 has been shown to have a large repertoire of substrates and can activate other proteinases [36]. Therefore, MMP-2 could potentially contribute to tumor progression via other mechanisms by processing molecules such as insulin like growth factor binding proteins (IGF-BPs), ephrin receptors and growth factors that contribute to angiogenesis [37], [38], [39], [40], [41]. However, our *in vitro* and *in vivo* studies suggest that MMP-2 processing of TGFβ is the principal mechanism underlying our observations. Furthermore, since the MMP-2 null animals used in the current study were systemically null for MMP-2, it is possible that MMP-2 derived from cellular sources other than the osteoblasts could regulate TGFβ activation. For example, the metastatic cancer cells in a number of human breast to bone metastases analyzed in our study were positive for MMP-2 and previously, the forced overexpression of MMP-2 or the endogenous tissue inhibitor of metalloproteinase-2 (TIMP-2) in cancer cells has been shown to promote or protect against tumor induced bone destruction respectively [42], [43]. Whether these effects are mediated via the control of TGFβ bioavailability is not known. These observations documenting the contribution of MMP-2 to cancer progression are not limited to bone since other studies have defined roles for MMP-2 in primary and metastatic tumor progression [41], [42]. Therefore, the generation of highly selective MMP-2 inhibitors could be useful for the treatment of a number of cancers including bone metastases. To this end, studies show that an MMP-2/-9 selective inhibitor, SB-3CT, significantly halts tumor progression in the bone [44]. We and others have previously shown that the ablation of host MMP-9 has a minimal impact on tumor/growth or tumor induced osteolysis [18], [45] thus implicating the importance of MMP-2 in the progression of bone metastases.

### MMP-2 processing of LTBP-3 potentiates TGFβ activation

Multiple studies have focused on the role of TGFβ in the tumor-bone microenvironment but often these studies have not examined the mechanisms of TGFβ activation. TGFβ is sequestered in a latency complex comprised of LAP and LTBPs. These complexes must be sequentially processed in order to generate active TGFβ [46], [47], [48], [49]. Interestingly, LTBP-3 null mice demonstrate altered skull development, osteoarthritis and osteopetrosis, defects that phenocopy those observed in animals with impaired TGFβ signaling in osteoblasts [50]. Our study identifies for the first time that osteoblast-derived MMP-2 is capable of processing LTBP-3 (Fig. 6E), and based on studies identifying MMP-2 as being able to process LAP-TGFβ [26], [51], we posit that MMP-2 subsequently cleaves LAP-TGFβ to release active TGFβ (Fig. 8C). Other members of the LTBP family such as LTBP-1 and 4 are MMP-2 substrates and LTBP-1 has been shown impact osteoblast mediated bone formation [24], [36], [47]. However, in mice, LTBP-1 deficiency results in a heart defect while LTBP-4 deficiency results in impaired lung development and colon tumorigenesis [52], [53], [54], [55] suggesting that LTBP-3 may be a key regulator of TGFβ bioavailability in the bone microenvironment. Based on our preliminary observations regarding MMP-2 processing of LTBP-3, we hypothesize that osteoblast derived MMP-2 is a key mediator of TGFβ activation in the tumor-bone microenvironment. It is important to note that MMP-9 and plasmin are also capable of processing TGFβ latency complexes thus potentially explaining why residual levels of active TGFβ were identified in the conditioned media derived from MMP-2 null osteoblasts (Fig. 6D) and also why TGFβ neutralizing antibody treatment with MMP-2 null conditioned media could further reduce the number of tumor colonies (Fig. 7B). However, given the data presented herein, plasmin and MMP-9 do not appear to compensate for the loss of MMP-2. In addition, while MMP-2 can directly mediate the activation of TGFβ, it may also initiate proteolytic cascades that ultimately lead to TGFβ activation [56], [57]. Conversely, MMP-2 is secreted in an inactive state and therefore, proteinases that can activate MMP-2 are indirectly capable of regulating TGFβ bioavailability and tumor survival. For example, MMP-14 has been shown to regulate MMP-2 activation and given the role of MMP-14 in skeletal development it is highly likely that osteoblast derived MMP-14 may impact the osteolytic vicious cycle by controlling MMP-2 activity [12], [58].

### MMP-2 and TGFβ effects on the osteoclast compartment of the vicious cycle

Our data demonstrate that osteoblast derived MMP-2 regulation of TGFβ bioavailability is critical for promoting tumor cell survival. While osteoclast precursors derived from MMP-2 null mice have no deficiencies with respect to migration and osteoclastogenesis, we cannot rule out that active TGFβ generated by osteoblasts may also have an effect on the osteoclast compartment of the vicious cycle. TGFβ has been shown to have differential effects on osteoclast behavior ranging from promoting osteoclast survival to osteoclastogenesis while some reports demonstrate that TGFβ can induce osteoclast apoptosis [59], [60]. These differential effects of TGFβ on osteoclast behavior may be dependent on the experimental conditions used in various studies. Thus, the precise role for TGFβ in regulating osteoclast behavior *in vivo* currently remains to be determined.

Surprisingly, our studies have shown that although MMP-2 does not appear to be expressed by mature osteoclasts, MMP-2 null osteoclast precursors undergo osteoclastogenesis more efficiently than wild type controls. This result is in contrast to previous reports showing that osteoclastogenesis is significantly attenuated in MMP-2 null bone marrow cultures [16]. These opposing conclusions may be due to the purity of the starting cultures (CD11b^+ve^ myeloid cells vs. whole bone marrow isolates) and underscores the importance of bone marrow stromal cells such as osteoblasts, leukocytes and mesenchymal progenitor cells in regulating osteoclastogenesis. Interestingly, humans with a deficiency in MMP-2 also have heightened areas of osteolysis and it is tempting to speculate that MMP-2 activation of TGFβ may be important in controlling osteoclast activity in this setting [61].

In conclusion, this study demonstrates how an osteoblast derived proteinase, MMP-2, can significantly impact mammary tumor growth in the bone microenvironment by enhancing tumor survival suggesting the presence of a mini-vicious cycle between the cancer cells and osteoblasts that is independent of osteoclast activity. We suggest that MMP-2 contributes to tumor survival by controlling the bioavailability of TGFβ via the processing of LTBPs, such as LTBP-3. Finally, our results support the rationale for the development of selective MMP inhibitors and/or the use of therapies that interfere with TGFβ signaling for the treatment of osteolytic breast to bone metastases.

## Materials and Methods

### Ethics Statement

All experiments involving animals and, primary cell lines isolated from animals (osteoclasts and osteoblasts), were conducted after review and institutional animal care and use committee (IACUC) approval by the office of animal welfare at Vanderbilt University (M/10/277 to CCL) and the Moffitt Cancer Center (3886R to CCL). De-identified human samples of frank osteolytic breast to bone metastasis (n = 11) and giant cell tumor (GCT, n = 1) were collected by curettage with IRB approval (IRB #40194) at Vanderbilt University from 2005 to 2010 with the patient's written consent.

### Reagents

Two different syngeneic FVB mammary tumor cell lines derived from the mammary tumor virus (MMTV) long terminal repeat-polyoma middle-T antigen (MMTV-PyMT) model of mammary tumorigenesis were isolated in our laboratory and maintained as previously described [19], [20]. These tumor cells lines were tagged with a luciferase reporter gene and designated, PyMT-Luc and 17L3C-Luc. All reagents were obtained from Sigma-Aldrich (St. Louis, MO, USA) except where specified.

### Histology

Fresh human breast-to-bone metastases and tumor and sham injected mouse tibiae were fixed overnight in 10% buffered formalin and decalcified for 3 weeks in 14% EDTA at pH 7.4 at 4°C with changes every three days. Tissues were dehydrated through ethanols, embedded in paraffin and 5 µm thick sections were cut. For MMP-2 localization (anti-MMP-2, AB37150, Abcam, Cambridge MA, USA), osteocalcin (anti-osteocalcin, sc-30044, Santa-Cruz Biotechnology, Santa-Cruz, CA, USA) a marker for used for osteoblasts [62], tumor proliferation (anti-Mcm2, AB31159, Abcam) and tumor apoptosis (anti-Caspase-3, #9662, Cell Signaling Technology, Danvers, MA, USA), the following technique was employed. Sections were rehydrated through a series of ethanols and then washed in Tris buffered saline with Tween-20 (TBST) (TBS; 10 mM Tris at pH 7.4, 150 mM NaCl with Tween-20 (0.05%). Following washing in TBS, tissue sections were blocked using standard blocking criteria for 1 hour at room temperature. MMP-2 (1∶150 dilution), osteocalcin (1∶100), Mcm2 (1∶200 dilution) and cleaved caspase-3 (1∶400 dilution) antibodies in blocking solution were added to the tissue sections and incubated overnight at 4°C. The appropriate IgG control antibodies were used for each antibody to ensure specificity. Slides were washed extensively in TBST prior to the addition of a species-specific secondary biotinylated IgG antibody (Vector Laboratories, Burlingame, CA, USA) diluted 1∶1,000 in blocking solution for 1 hour at room temperature. Labeled cells were visualized using an avidin-biotin peroxidase complex (Vectastain ABC kit, Vector Laboratories) and 3,3′ –Diaminobenzidine tetrahydrochloride substrate. Sections were counterstained with hematoxylin prior to dehydration through ethanols and permanently mounted.

Tartrate-resistant acid phosphatase (TRAcP), a marker of mature osteoclasts, was detected using a colorimetric kit according to the manufacturer's instructions (Kit 387-A, Sigma-Aldrich) or via immunohistochemistry as described (18-0199, Invitrogen). Gross anatomy of the mouse tibiae was assessed by hematoxylin and eosin (H&E) staining. Immunofluorescent localization of MMP-2, osteocalcin and TRAcP assays were performed as previously described [18], [63].

### Intratibial injection and *in vivo* quantitation of tumor growth

PyMT-Luc or 17L3C-Luc tumor cells (2×10^5^) in a 10 µl volume of sterile phosphate buffered saline (PBS) were injected into the tibia of anesthetized immunocompetent 6 week old female mice that were wild type or null for MMP-2. The contralateral limb was injected with 10 µl of PBS alone and acted as a sham injected control for changes in the bone due to the surgical procedure. The IVIS™ system (Caliper Life Sciences, Hopkinton, MA) was used to detect luminescence from the PyMT-Luc and 17L3C-Luc tumor cells after intratibial injection. Firefly luciferin (120 mg/kg in sterile PBS, Gold Biotechnology, Inc., St. Louis, MO) was delivered retro-orbitally 2 minutes prior imaging. Mice were imaged at 24 hours and every 3 days after surgery. Living Image™ software (Caliper Life Sciences) was used to quantify the luminescence intensity in the tumor-bearing limb over time. For the histology and histomorphometry studies, mice were sacrificed at 9 days post-surgery which was previously determined to be the time point prior to tumor breach of the cortical bone by PyMT-Luc in wild type control mice [19]. For immunohistochemical staining, mice injected with tumor cells were collected at described time points and both tumor bearing and control tibias were collected. All animal studies were independently repeated on five independent occasions.

### Micro computed tomography (μCT), x-ray radiography and histomorphometric analyses

For gross analysis of trabecular bone volume, formalin fixed tibiae were scanned at an isotropic voxel size of 12 µm using a microCT40 (SCANCO Medical, Bruttisellen, Switzerland). The tissue volume (TV) was derived from generating a contour around the metaphyseal trabecular bone that excluded the cortices. The area of measurement began at least 0.2 mm below the growth plate and was extended by 0.12 mm. The bone volume (BV) included all bone tissue that had a material density greater than 438.7 mgHA/cm^3^. These analyses allowed for the calculation of the BV/TV ratio. The same threshold setting for bone tissue was used for all samples. Radiographic images (Faxitron X-ray Corp, Lincolnshire, IL, USA) were obtained using an energy of 35 kV and an exposure time of 8 seconds. The tumor volume (TuV) was calculated as a function of the total tissue volume (TV) of the tibial medullary canal using Metamorph® software (Molecular Devices, Sunnyvale, CA, USA).

For histomorphometry, three non-serial sections of tumor bearing and sham injected hind limbs were H&E stained to assess the BV/TV ratio or with TRAcP to assess osteoclast number per mm bone at the tumor bone interface using Metamorph® software (Molecular Devices).

### Isolation of osteoclast precursor cells and primary osteoblasts

All primary cell lines were collected from mice with IACUC approval (Vanderbilt University (CCL-M/10/277) and Moffitt Cancer Center (CCL-3886R)). For the collection of osteoclast precursor cells, the bone marrow from the tibias and femurs of 6 week-old wild type and MMP-2 null mice were collected by flushing the cells with 1 ml of cold 1× PBS using a 25G^5/8^ gauge needle. Isolated cells were centrifuged at 1,000 rpm and rinsed with 1 ml of 1× PBS. CD11^+ve^ cells were then isolated using a Macs® separation protocol as per the manufacturer's directions (Cat No. 130-049-601, Miltenyi Biotec, Auburn, CA, USA) and plated in α-MEM and 10% fetal bovine serum (Cat. No. 0500A, Atlas Biologicals, Fort Collins, CO, USA), 100 µg/ml penicillin/streptomycin with 250 UG/ml fungizone (Invitrogen) for migration and differentiation assays.

For osteoblast primary cultures, calvaria from wild type or MMP-2 null 3 day-old neonates were isolated in cold sterile 1× PBS buffer. Calvaria were then subjected to three repetitive digestions (15 min, 30 min and 1 hour incubations) in digestion buffer (α-MEM, Cat. No. 12571, 0.025% trypsin, Cat. No. 25200, 100 µg/ml penicillin/streptomycin Cat. No. 15070-063, 250 UG/ml fungizone Cat. No. 15290-018, Invitrogen and 10 mg/ml collagenase P, Cat. No. 11213857001, Roche, Basel, Switzerland) at 37°C with vigorous shaking every 15 min. Isolated primary cells were then maintained in α-MEM and 10% fetal bovine serum (Atlas Biologicals). Primary osteoblasts were plated at a density of 2×10^5^ cells/well in 6 well-plates and 24 h after seeding; cells were cultured in serum-free α-MEM. After 24 h, conditioned media was collected, centrifuged at 1100 rpm to remove cellular debris and used for the MTT and soft agar colony formation assays described below.

### Migration assay of osteoclast precursor cells

CD11b^+ve^ cells were plated at a density of 10^5^ cells/well in the upper well of a transwell (5 µm pore size, Corning Inc., Corning, NY, USA) in 500 µl of serum free α-MEM media. Cells were allowed to migrate towards the lower well of the transwell (1 ml of chemotactic gradient (10% serum-α-MEM) or serum-free media as control) for 5 hours at 37°C. CD11b^+ve^ that migrated through the membrane were harvested in the lower well and counted. Experiments were performed in triplicate.

### Differentiation of osteoclast precursor cell assay

CD11b^+ve^ cells isolated from 6 week-old wild type and MMP-2 null bone marrow cells were plated in 48 well plates in 10% serum-α-MEM media at a density of 5×10^5^ cells/well. The following day, cells were treated with 75 ng/ml RANKL (R&D Systems, Minneapolis, MN, USA) and 30 ng/ml M-CSF (R&D Systems) in 500 µl of 10% serum-α-MEM media. Media was changed every 3 days for a 15 day period. At the end of the assay, cells were fixed in ice-cold methanol and stained using a colorimetric TRAcP kit (Sigma-Aldrich) and counter stained in hematoxylin. Multinucleated (more than 3 nuclei) TRAcP cells were counted in eight random fields acquired using a 10× microscopic objective for each condition. Experiments were performed in quadruplicate. For osteoclast functionality assays, dentin discs were removed from culture and sonicated for 2 min in 5 ml of 0.25 M ammonium hydroxide to remove cells. The discs were then stained for 5 min (0.05% toluidine blue in 40% MeOH) and air-dried. The total number of pits formed per disc was counted using reflective light microscopy.

### Osteoblast characterization and zymography

Primary osteoblasts were cultured for two weeks in the presence or absence of osteogenic media (OGM; 10 mM biglycerol phosphate, 50 µM ascorbic acid, 25 ng/ml bone morphogenetic protein-2 (BMP-2; R&D Systems) in 10% serum containing alpha MEM). After 2 weeks of incubation, the cells were assessed for alkaline phosphatase activity as a readout for differentiation. Osteoblast cell lysates were generated using standard lysis buffers. The total protein content in the cells was measured by BCA assay and alkaline phosphatase activity was measured in normalized samples using p-nitrophenyl phosphate (p-NPP; Pierce, Rockford, IL, USA) in a 1 M diethanolamine buffer at pH 9.8. Absorbance in control and OGM treated cells was measured at 405 nm.

For analysis of MMP-2 enzymatic activity, wild type and MMP-2 null primary osteoblast cultures were seeded at a concentration of 5×10^5^ cells per 60 mm dish. After 48 hours incubation, the cells were incubated in serum free media for 3 hours. Afterwards, the cells were rinsed in 1×PBS and incubated for 24 hours in 2.5 ml of serum free media. Subsequently, the total protein in the collected conditioned media was measured by BCA (Pierce) assay and the samples were normalized for total protein concentration prior to zymography (25 µg total protein per well). For gelatin zymography, gelatin was added to SDS resolving gels to a final concentration of 1 mg/ml and equal amounts of total protein were run under non-reducing conditions. After electrophoresis the gels were washed in 2.5% solution of Triton-X-100 followed by overnight incubation in substrate buffer (50 mM Tris-HCL, pH 7.4 containing 5 mM CaCl2). The following day, the gels were staining in a 5 mg/ml coomassie brilliant blue solution (1 part acetic acid: 3 parts isopropyl alcohol: 6 parts water). The gels were then destained in water and digitized.

### MTT Assay

Quantitation of viable PyMT-Luc cells treated with conditioned media from primary osteoblast wild-type or MMP-2 null mice was assessed by tetrazolium-based colorimetric MTT assay (Cat. No. G5421, Promega, Madison, WI, USA). Tumor cells were plated in 96-well plates at a density of 1000 cells/well and 24 h after seeding, cells were treated with 100 µl either serum-free or conditioned media from primary osteoblasts isolated from either wild type or MMP-2 null mice. After 24 h of treatment, 20 µl of MTS was added to each well, and the reactions were allowed to run for 3 h at 37°C. Spectrophotometric absorbance of each sample was measured at 490 nm using a MRX revelation microplate reader (Dynex Technologies, Chantilly, VA). Experiments were performed in quadruplicate.

### Expression and enzymatic processing assays

COS-7 cells were transiently transfected with a full length LTBP-3 cDNA construct and human TGFβ1 cDNA (kindly provided by Dr David Rifkin, [32] using Superfect kit (QIAGEN, Carlsbad, CA, USA). COS-7 cells were plated at a density of 10^5^ cells/well in a 6 well plate the day prior the transfection. Cells were then incubated in transfection mix (30 µl of Superfect reagent, 0.5 µg of each constructs and 500 µl of DMEM and 10% fetal bovine serum) overnight. The next day, transfected COS-7 cells were incubated for 48 hours in serum starved DMEM media. The conditioned media was then incubated for 3 hours in presence of 300 ng of recombinant human MMP-2 or for 1 hour in presence of 1 µg plasmin (Sigma-Aldrich) as a positive control [35]. Samples were then analyzed by immunoblotting for LTBP-3 as described below.

### Immunoblotting and ELISA

Tumor and sham injected tibias from wild type or MMP null animals were harvested 3 days post-injection and flash frozen in liquid nitrogen. Tissue homogenates were generated by mortar and pestle and total protein was subsequently extracted using a standard protein lysis buffer (0.1% sodium dodecyl sulfate, 0.5% sodium deoxycholate, 1% triton X100, 10 mM Tris pH 7.5, 150 mM NaCl) containing a complete proteinase inhibitor cocktail (Roche) and phosphatase inhibitor cocktails (Sigma-Aldrich). Protein concentration in isolated samples was quantitated using a bicinchoninic acid (BCA) assay as per manufacturer's instructions (Pierce). Equal concentrations of total protein were loaded on to a denaturing 8% SDS-PAGE gel. The blots were then panned with antibodies directed to, phospho Smad2 (Millipore), total Smad2 (Cat. No. 3102, Cell Signaling Technology), phospho AKT and AKT (Cell Signaling Technology) and actin (Santa Cruz Biotechnology, Santa Cruz, CA, USA). All antibodies were diluted 1∶ 1,000 in 5% BSA in 1×TBST (TBS with 0.05% Tween 20) overnight with rocking at 4°C. For latent binding protein-3 (LTBP-3) immunoblotting, equal amounts of protein were loaded on 6% SDS-PAGE non-denaturing gels. Blots were then incubated anti-LTBP-3 antibodies (anti-L3C [32]) diluted 1∶ 1,000 in standard blocking solution (5% milk in 1× TBS) overnight at 4°C with rocking. The following day, blots were washed extensively with 1×TBST prior to the addition of a secondary infra-red labeled antibody (1∶ 5,000 dilution in 1×TBST, Rockland Inc., Gilbertsville, PA, USA) for 1 hour at room temperature with rocking, in the dark. After washing in 1×TBST, blots were developed and bands of interest were quantitated using the Odyssey system (LI-COR Biosciences, Lincoln, NE, USA). ELISA assays for TGFβ (Cat. No. MB100B, R&D Systems) were performed according to the manufacturer's instructions.

### Soft agar colony formation assay

PyMT-Luc cells were plated at a density of 1.5×10^3^ cells/well in 24 well-plates in soft agar containing α-MEM, 5% fetal bovine serum, 0.7% agarose (Cat. No. BP164, Fisher Scientific, Fair Lawn, NJ, USA). Subsequent to plating, tumor cells were treated with 400 µl of either with 5% serum α-MEM or conditioned media derived from either wild type or MMP-2 null primary osteoblasts supplemented with 5% serum. Experimental conditions also included the addition of the TGFβ neutralization antibody 2G7 at 10 µg/ml or an IgG2b control antibody [64] in the presence or absence of 100 ng/ml recombinant active MMP-2 (Cat. No. PF023, EMD Biosciences, San Diego, CA). Soft agar PyMT-Luc colony formation assays were also performed with 1 ng/ml TGFβ in 5% serum α-MEM. The media with various experimental conditions was changed every 3 days. After 10 days of culture, cells were stained overnight with 0.1 mg/ml p-iodonitrotetrazolium (Cat. No. I-8377, Sigma-Aldrich). Numbers of colonies and average diameter of the colonies for each condition were measured on 100× photomicrographs and analyzed using Meta morph Imaging Software (Molecular Devices). Experiments were performed in quadruplicate.

### 2D-Colony formation assay

PyMT-Luc were seeded into 24 well plates (50 cells per well). The cells were allowed to attach and then treated with 400 µl of α-MEM (Control) or conditioned media derived from either wild type or MMP-2 null primary osteoblasts all supplemented with 5% serum. Experimental conditions also included the addition of the TGFβ neutralization antibody 2G7 at 10 µg/ml or an IgG2b control antibody. The media was changed every 3 days for 10 days. To assess colony formation, wells were rinsed in 1×PBS then the colonies were fixed and stained in a solution containing 6% glutaraldehyde and 0.5% crystal violet for 30 minutes at room temperature. The wells were rinsed twice in dH2O prior to drying and counting the total number of colonies per well.

### Statistical analyses

Statistical analyses were performed using Student's *t* Test or ANOVA where appropriate using GraphPad Prism (GraphPad Software, Inc., La Jolla, CA, USA). A value of p<0.05 was considered significant. Data are presented as mean ± standard deviation (SD).
